# Long-Term Trends and Role of Climate in the Population Dynamics of Eurasian Reindeer

**DOI:** 10.1371/journal.pone.0158359

**Published:** 2016-06-30

**Authors:** Alessia Uboni, Tim Horstkotte, Elina Kaarlejärvi, Anthony Sévêque, Florian Stammler, Johan Olofsson, Bruce C. Forbes, Jon Moen

**Affiliations:** 1 Department of Ecology and Environmental Science, Umeå University, Umeå, Sweden; 2 Department of Geography and Geology, University of Turku, Turku, Finland; 3 Plant Biology and Nature Management, Vrije Universiteit Brussel, Brussels, Belgium; 4 Arctic Centre, University of Lapland, Rovaniemi, Finland; University of Colorado, UNITED STATES

## Abstract

Temperature is increasing in Arctic and sub-Arctic regions at a higher rate than anywhere else in the world. The frequency and nature of precipitation events are also predicted to change in the future. These changes in climate are expected, together with increasing human pressures, to have significant impacts on Arctic and sub-Arctic species and ecosystems. Due to the key role that reindeer play in those ecosystems, it is essential to understand how climate will affect the region’s most important species. Our study assesses the role of climate on the dynamics of fourteen Eurasian reindeer (*Rangifer tarandus*) populations, using for the first time data on reindeer abundance collected over a 70-year period, including both wild and semi-domesticated reindeer, and covering more than half of the species’ total range. We analyzed trends in population dynamics, investigated synchrony among population growth rates, and assessed the effects of climate on population growth rates. Trends in the population dynamics were remarkably heterogeneous. Synchrony was apparent only among some populations and was not correlated with distance among population ranges. Proxies of climate variability mostly failed to explain population growth rates and synchrony. For both wild and semi-domesticated populations, local weather, biotic pressures, loss of habitat and human disturbances appear to have been more important drivers of reindeer population dynamics than climate. In semi-domesticated populations, management strategies may have masked the effects of climate. Conservation efforts should aim to mitigate human disturbances, which could exacerbate the potentially negative effects of climate change on reindeer populations in the future. Special protection and support should be granted to those semi-domesticated populations that suffered the most because of the collapse of the Soviet Union, in order to protect the livelihood of indigenous peoples that depend on the species, and the multi-faceted role that reindeer exert in Arctic ecosystems.

## Introduction

Terrestrial ecosystems in the Arctic and sub-Arctic are adapted to low-temperature regimes and therefore considered to be extremely susceptible to rapid climate change, especially because temperature increase in these regions has been two to three times higher than in other parts of the world in recent decades [1]. Moreover, in many parts of the Arctic precipitation is also expected to increase, potentially in the form of snow and rain-on-snow [2]. The latest assessment of changes in NDVI (Normalized Difference Vegetation Index, a proxy for plant productivity) from satellite observations between 1982 and 2012 shows that about a third of the Pan-Arctic has substantially greened [1,3]. However, drivers of low Arctic tundra greening, where it is occurring, can be diverse even over distances of only a few dozen kilometers [3]. Herbivores are one such driver since they can counterbalance the effects of climate change on Arctic greening by limiting the growth of plants that would otherwise flourish at higher temperatures, such as shrubs and forbs [4,5].

*Rangifer tarandus* (including both reindeer and caribou, but hereafter referred to as reindeer) is the herbivore species with the highest potential to prevent greening in the Arctic. The species has a circumpolar distribution. While in North America the species persists mainly in wild populations, in Eurasia reindeer husbandry evolved thousands of years ago and nowadays both wild and semi-domesticated populations can be found [6]. As of 2010, the Arctic and sub-Arctic regions hosted approximately 3.8 million wild reindeer and caribou and 2 million semi-domesticated reindeer [7]. The livelihood of at least 20 indigenous peoples around the circumpolar North revolves around reindeer [8–11]. Most of the semi-domesticated reindeer populations feed on natural resources year-round and are therefore integral to the functioning of Arctic social-ecological systems [6].

Due to the importance of reindeer in mediating potential feedbacks to climate change and shaping Arctic plant and wildlife communities and human culture, it is crucial to assess to what extent climate affects reindeer populations. Climate change may affect reindeer both negatively and positively. Reindeer migrate from winter to summer pastures following plant phenology. Earlier and warmer springs may advance phenology and, if reindeer do not adapt the timing of their migrations to the new temporal and spatial patterns of vegetation seasonal growth, a mismatch may occur between plant phenology and herbivore presence [12]. Moreover, warming may increase the occurrence of ice-crust formation on winter pastures. In winter, reindeer are highly dependent on the availability of ground lichens, which constitute their primary source of food [13]. Ground lichens may become unavailable when warm temperatures melt the snow on the ground and then subsequent cold temperatures freeze it to form an ice crust. Similarly, freezing rain and rain on snow can also form a thick ice layer that reindeer cannot penetrate (reviewed in [14]). Warmer summers may also increase insect harassment, decreasing the amount of time that reindeer allot to feeding (reviewed in [15]). On the other hand, an increased length of the growing season may increase forage quantity and quality during summer, leading to positive effects on reindeer survival and reproductive success (reviewed in [15]). Our knowledge of the effects of climate on reindeer population dynamics comes from studies that focus either on short intervals of time (e.g., [16]) or on small spatial scale (e.g., [17,18]). We are lacking a comprehensive view of the effects of climate on reindeer population dynamics, i.e. we do not know if the results from local studies conducted over short periods can be extrapolated to longer periods and larger spatial scales.

The scope of our study was to examine the effects of climate on reindeer population dynamics, using for the first time long-term datasets (covering a period up to 70-year long) collected from more than half of the species’ circumpolar range (from Fennoscandia to Far Eastern Siberia). As mentioned above, semi-domesticated reindeer are an important part of Arctic and sub-Arctic ecosystems and often rely on natural forage like their wild counterpart [6]. Thus, we included data on both wild and semi-domesticated populations in our analysis.

In particular, our study aimed to: i) analyze the trends in reindeer population dynamics over the last 70 years; ii) evaluate if reindeer populations fluctuated in a synchronous manner; and iii) determine the influence of climate (represented by proxies of climatic variability) on yearly growth rates. We decided to include semi-domesticated reindeer in our study also because we aimed to assess if climate had the power to drive population dynamics despite all the measures taken by herders to protect their herds (e.g., supplemental feeding in adverse weather conditions). We tested both the direct and delayed effects of winter climate. A particularly good or bad winter could have direct effects on population growth rates via reindeer survival, or delayed effects through its impact on calf body condition and reproduction potential during following years [19].

Directional changes in climate on a global level, such as the ones we are witnessing today, are expected to affect in a synchronous manner the dynamics of populations belonging to the same species but living far from each other (a phenomenon defined as "Moran effect" [20,21]). In other words, if a climatic parameter (such as temperature) increases worldwide, we should observe synchronous increases or declines in the abundance of all/most populations of a species as a consequence of that abiotic change, if the climatic force outweighs local noise [22,23]. Therefore, we anticipated reindeer populations to manifest some level of synchrony in their dynamics. Because neighboring populations may experience similar changes in environmental conditions, as well as migration of individuals between populations, we expected synchrony to be correlated with the distance separating population ranges.

## Methods

### Reindeer abundance time series

**Fig 1:**
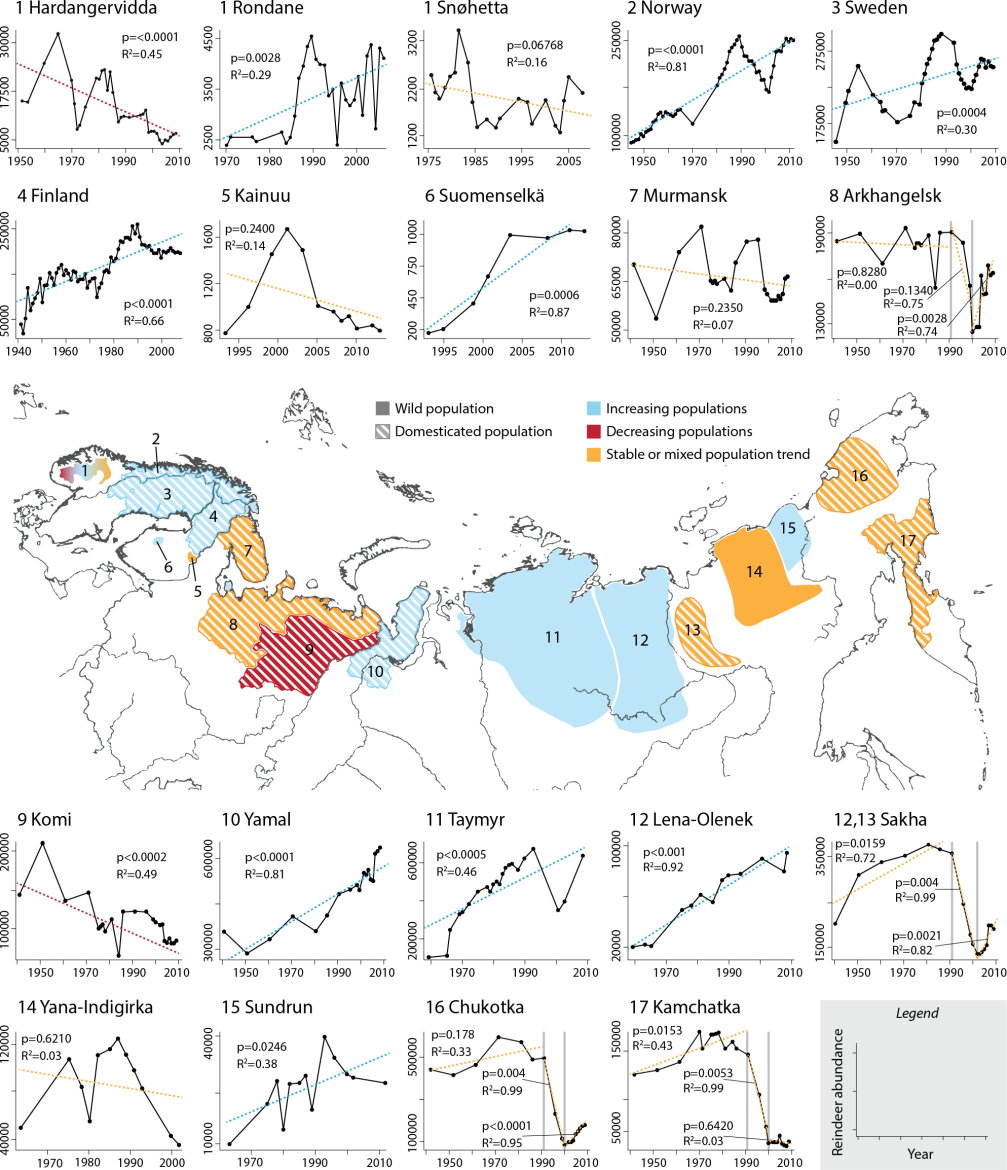
***Central panel*—Ranges of 19 major reindeer populations in Eurasia. *Upper and lower panels*—Plots representing the time series of available abundances for each population.** Each plot number corresponds to a range in the map and is followed by the name of the population. The color-coded lines in the plots show the trend in the time series (p-values and R^2^ provided for each trend are the result of regression analyses with time in years as predictor variable and reindeer abundance as response variable).

We gathered time series of annual counts of 19 reindeer populations (n = 9 wild; n = 10 semi-domesticated), ranging from Fennoscandia to Far Eastern Siberia (Fig 1). We only selected populations for which abundance data were available until at least the year 2000, to be able to capture reindeer response to climate in recent decades. For most populations, the spatial scale of analysis was large (e.g. country level for semi-domesticated populations in Fennoscandia and district level, i.e. okrug, in Russia). However, data on wild populations in Fennoscandia were available only on a smaller spatial scale, i.e. at the herd level, and temporal mismatch among abundance time series of those herds did not allow us to combine them at the country level. Data sources are detailed in S1 Table. Number of years with available data and length of each time series are described in S2 Table.

All abundance data from Scandinavian semi-domesticated populations come from annual counts conducted by the herders during slaughter round-ups and represent the size of the winter herd (S1 Table). In Norway, counts are checked by authorities and the error was estimated as being < 10% [24]. Alaruikka [25] estimated that before 1964 ~15% of all reindeer escaped annual round-ups in Finland. In the late ‘70s and early ‘80s, the proportion of ear-marked yearlings missing in each round-up in Finland was 1–2% [26]. Accuracy measures are not available for Swedish and Russian semi-domesticated populations.

Tundra dwelling wild reindeer populations in Russia were counted from aircraft during the warmest days in July-August, when animals aggregate to escape insect harassment [27–30]. Accuracy of these datasets varies between years depending e.g. on weather and economic conditions [29], but data are accurate enough to analyze long-term trends [27]. According to Kolpasсhikov et al. [28], herd size in Taymyr has a confidence interval varying between ± 5% and ± 10% depending on weather conditions. However, after 1991 wild reindeer numbers in Russia may have been underestimated because of reduced counting efforts compared to the previous period [28,31].

### Climate and weather data

The time series of three indices serving as proxies of ocean-atmosphere climatic variability (hereafter defined as climate indices) were used: the North Atlantic Oscillation (NAO), the Arctic Oscillation (AO) and the North Pacific (NP) index. Only winter values (i.e. January to March) for these indices were considered, since the atmospheric circulations over the North Pacific and North Atlantic oceans are strongest during winter [32,33]. The time series of the principal component (PC)-based NAO index [32] was downloaded from the Climate Data Guide (https://climatedataguide.ucar.edu/climate-data/hurrell-north-atlantic-oscillation-nao-index-pc-based; accessed on October 1^st^, 2013). The NAO winter (January–March) index represents the annual deviation during winter from the average difference in sea-level atmospheric pressure between Lisbon (Portugal) and Stykkisholmur (Iceland), after applying a normalization in relation to the time range 1864–1994 [32]. This index is available starting in 1899. The circulation over the Atlantic Ocean expressed by the NAO has an influence over Europe and further east toward Asia [34]. In years when the NAO is positive, winds blowing over the Atlantic transport warm and moist air masses to Northern Europe, resulting in warm and wet winters. In contrast, cold and dry winters prevail during years with negative NAO values. The AO time series was retrieved from the National Weather Service (http://www.cpc.ncep.noaa.gov; accessed on October, 1^st^ 2013) and annual winter values were averaged from the winter monthly values (January–March). The AO time series starts in 1950. During the positive phase of the AO, cold air is trapped over the North Pole leading to warmer temperatures in Northern Europe and Siberia. The AO index is highly correlated to the NAO (Pearson's r = 0.90 during our study period, [35]), but we included both indices in our analysis for comparison with previous and future studies. Indeed, both indices have been shown to correlate with population dynamics of some reindeer populations in Eurasia (e.g., see [17,36,37]). Finally, the NP index [33] was retrieved from the Climate Data Guide (https://climatedataguide.ucar.edu/climate-data/north-pacific-np-index-trenberth-and-hurrell-monthly-and-winter; accessed on October 1^st^, 2013). During the positive phase of the NP, the air pressure at sea-level is higher than normal in the North Pacific, leading to higher than normal precipitations on the east coast of Asia and warm temperatures in the western North Pacific [38]. We expected the NP index to be a predictor of reindeer population dynamics in Far Eastern Siberia.

We retrieved mean regional temperatures from the weather station data provided by the Berkeley Earth Surface Temperature project (http://berkeleyearth.org/data; accessed on March 31^st^ 2014). We selected all the weather stations available in the range of each of the studied reindeer populations (between 4 and 20 weather stations per range after removing stations with too few data). We extracted the mean monthly temperatures for the whole study period (1941–2012), and averaged the mean temperatures of January, February, and March for each year. We obtained total winter precipitation by summing daily precipitation in the same winter months and averaging them within each reindeer population range. Russian precipitation data were obtained from the Carbon Dioxide Information Analysis Center website (http://cdiac.ornl.gov/ndps/russia_daily518.html; accessed on March 31^st^ 2014), which provided data for 518 Russian weather stations [39]. Stations were excluded from the analysis in those years when more than 10% of daily precipitation data were missing for the January-March period. Norwegian precipitation data were obtained from the Norwegian Meteorological Institute (http://eklima.met.no; accessed on October 1^st^, 2014), while Swedish precipitation data were obtained from the Swedish Meteorological and Hydrological Institute (http://opendata-download-metobs.smhi.se/explore/#; accessed on October 1^st^, 2014). Due to restricted availability of the Finnish precipitation data, we did not include them in the analysis.

### Statistical analyses

#### Long-term trends in population dynamics

The first step of our analysis consisted in exploring the trends in the dynamics of each reindeer population using linear regression, with reindeer abundance as response variable and time (i.e., year) as predictor variable (following [40]).

During the study period (1941–2012), the areas occupied by reindeer populations in Russia underwent significant socio-economic changes. These changes strongly affected the dynamics of some semi-domesticated populations. In particular, during the Soviet Union time (i.e. until 1991) the government was promoting and supporting a meat-producing industry that aimed at maximizing production in the semi-domesticated populations, e.g. with economic incentives and subsidies. After the collapse of the Soviet Union in 1991, those subsidies ceased and reindeer herders in some communities could not afford to maintain large herds anymore. As a result, some regions in Russia experienced a strong collapse in semi-domesticated reindeer numbers in the 1990s [41,42], which is evident in the abundance time series of those populations (Fig 1). Therefore, we ran separate linear regression models through each section of the time series (our choice of breakpoints is supported by [43]).

#### Synchrony among population growth rates

When analyzing synchrony in population dynamics, we were interested in synchrony in the short-term fluctuations of pairs of populations. These patterns may be hidden by synchrony in long-term trends. To exclude long-term synchrony and concentrate on the short-term patterns, synchrony should be estimated based on population growth rates rather than population abundances [20]. Therefore, we used reindeer abundances to estimate population growth rates as the first-differenced time series of log-abundances (ln(*N_t_*) − ln(*N*_*t*−1_), where N_t_ = population abundance at time t). Then, we calculated synchrony of growth rates between pairs of populations, using zero-lag cross-correlation (i.e. pairwise Pearson correlation coefficients, [22]). Thus, high statistically significant correlation coefficients indicate a high level of synchrony between population growth rates. We used a non-parametric bootstrapping procedure based on 1000 resampling draws with replacement to calculate the 95% confidence interval of each pairwise Pearson correlation coefficient. The resampling with replacement was performed on the growth rates of each time series (i.e., Method III in [44]), since we did not detect any temporal autocorrelation among subsequent growth rates. For each pair of populations, we considered only those years when we had data for both populations.

Because of the possible underestimation in the counts conducted after 1991 in the Russian wild reindeer ranges [28,31] and the contrasting opinions on survey reliability from those years found in the literature [27,28], we decided to disregard data collected after 1991 in the wild Russian populations for the synchrony analysis. This procedure reduced the sample size to 11 counts for the Lena-Olenek population, 9 for Yana-Indigirka, 8 for Sundrun, and 18 for Taymyr. Sample sizes for three out of four populations thus became too small to perform the statistical analysis. Therefore, we removed the Lena-Olenek, Yana-Indigirka, and Sundrun populations from the synchrony analysis. We also excluded the two wild Finnish populations (Kainuu and Suomenselkä) because of the short time frame of available counts (S2 Table). For the Taymyr population, we truncated the analysis at the count conducted in 1990.

Synchrony among neighboring populations may be driven by spatially correlated changes in environmental conditions or by migration of individuals between populations. To test if neighboring populations experienced more synchronous dynamics than distant populations, we assessed the relationship between synchrony in population growth rates and the linear distance between centroids of population ranges.

#### Linking climatic variability to population growth rates

To test if climatic variability influenced reindeer population growth rates, we ran linear regression models for each population with population growth rate as dependent variable and a climate index as predictor variable (i.e., NAO, AO and NP analyzed individually in separate models). Population growth rates were calculated as *r* = ln(*N_t_*/*N*_*t*−1_), where N_t_ = population abundance at time t. Because of gaps in the abundance time series, we averaged r and the climate indices over periods with missing abundance estimates (following [45]). For example, if population abundances were available only for the years 1981 and 1986 but not for the years in between, we calculated the population growth rate from 1981 to 1986 (which is the sum of the yearly growth rates in that period) and then averaged both the growth rate and the climate indices across the winters included in that time range. We also tested for one-year and two-year delayed effects of climate by shifting the climate index averages one or two years back in time. Our models did not contain temporal autocorrelation of the residuals, so no correlation structure was necessary. We checked all models for violations of regression assumptions (heteroscedasticity and non-normality of the residuals) but did not find any. We used the Bonferroni-adjusted outlier test in the R package “car” [46] to detect outliers and we removed them if present. As for the synchrony analysis (see above), we excluded the Lena-Olenek, Yana-Indigirka, Sundrun, Kainuu, and Suomenselkä populations.

The crucial socio-economic changes that followed the collapse of the Soviet Union in 1991 possibly confounded the effects of climate in Russia. Therefore, we ran multiple regression models for the Russian semi-domesticated populations. Those models included a climate index, a categorical variable and the interaction between these variables as predictors. If the categorical variable or the interaction were not statistically significant (p-value > 0.05), they were removed from the model. The climate index was always left in the model, since it was our predictor of interest. The categorical variable defined the periods before (1941–1990), during (1991–2000) and after (2001–2009) the collapse of the Soviet Union.

#### Relationship between climate indices and regional temperature and precipitation

The NAO, AO, and NP indices show spatial heterogeneity in their relationship with local weather [32,34,47], but a synthesis of how they correlate with temperature and precipitation in our whole study region is missing. Therefore, we calculated Pearson correlation coefficients between each winter climate index and mean winter (January-March) temperature and total winter precipitation in Fennoscandia and in the ranges occupied by the Russian reindeer populations (Fig 1).

All statistical analyses were conducted in R, version 3.0.2 (R Development Core Team [48]).

## Results

### Trends in population dynamics

The trends in the dynamics of the 19 reindeer populations we studied were very heterogeneous (Fig 1). Populations of semi-domesticated reindeer in Fennoscandia exhibited strong fluctuations in abundance, but were overall increasing over the last 70 years after an historical low reached in the 1940s. The two wild Finnish populations (Kainuu and Suomenselkä) exhibited very different trends, as did the three wild Norwegian populations (Hardangervidda, Rondane, and Snøhetta). Similarly, the Russian semi-domesticated populations in Komi and Yamal decreased and increased, respectively. Murmansk experienced considerable fluctuations, but without a clear trend over the study period. The other semi-domesticated Russian populations experienced a significant synchronous decline in the 1990s, after the Soviet Union collapse. Most of the wild Russian populations increased during the study period, except for the Yana-Indigirka population, which experienced a pronounced decrease over the last two decades.

### Population growth rate synchrony

**Fig 2:**
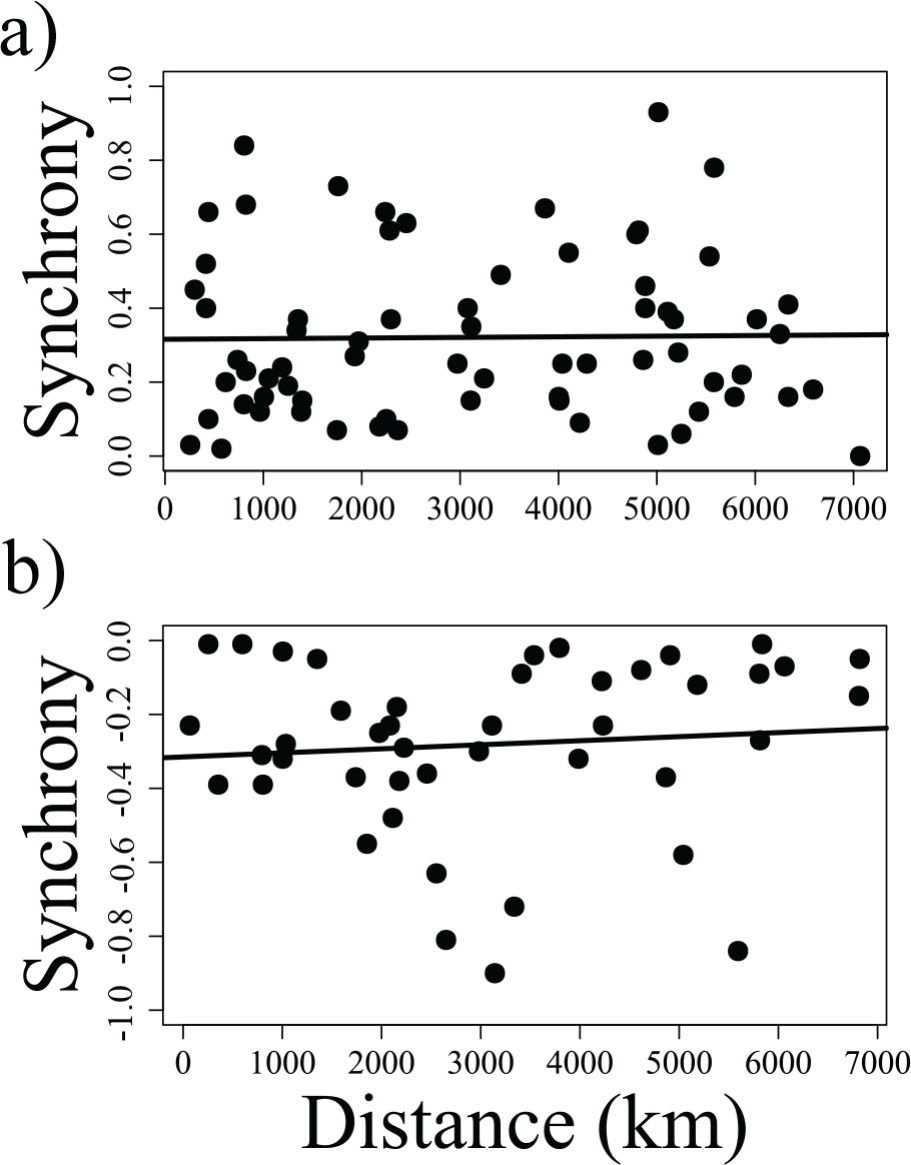
Relationship between synchrony in the growth rates of pairs of reindeer populations and the distance separating them. Distance was estimated as the linear distance separating centroids of population ranges. Panel a) represents all positive synchronies we detected; panel b) represents all negative synchronies we detected (see S3 Table for details). The solid, black lines represent the results of linear regression models with synchrony as response variable and distance as predictor variable (both relationships not significant, panel a): p-value = 0.9240; panel b): p-value = 0.7447).

Synchrony among population growth rates was overall low (Fig 2). Synchrony was stronger among semi-domesticated populations than among wild populations (S3 Table). Twelve of 42 pairs of semi-domesticated reindeer populations (29%) were significantly positively correlated. Among the six pairs of wild populations, none were significantly correlated. Two wild populations were negatively correlated to two semi-domesticated populations. We were unable to calculate synchrony for seven pairs of populations (8%) due to temporal mismatch between their abundance time series (see NAs in S3 Table). We did not detect any significant relationship between distance among population ranges and synchrony (Fig 2).

### Linking climatic variability to population growth rates

Climatic variability explained only a part of the variability in population growth rates of only few populations (Tables 1 and 2). Among the Scandinavian semi-domesticated and wild populations, the NAO and AO indices explained some of the variability in population growth rates of the Norwegian semi-domesticated population with a negative, one-year lagged effect. The AO index also explained part of the variability in the growth rates of the Hardangervidda wild population with a positive, two-year lagged effect (Table 1). The only Russian wild population we were able to analyze (Taymyr) was not affected by the climatic variability described by the indices we used. Among the Russian semi-domesticated populations (Table 2), the NAO index explained some of the variability in population growth rates of the Sakha population with a negative, two-year lagged effect. The AO index explained the dynamics of the Kamchatka population with a positive, two-year lagged effect. The NP index explained the dynamics of the Yamal population with a positive, one-year lagged effect, and the dynamics of the Kamchatka populations with a positive, two-year lagged effect. The effects of the NAO index (not lagged) in Murmansk and the NP index in Arkhangelsk (two-year lagged), Sakha (one-year lagged) and Chukotka (not lagged and one-year lagged) differed between the periods before, during and after the collapse of the Soviet Union. As expected, the NAO index explained mostly the variability in areas closer to the Atlantic Ocean and the NP index explained the variability in areas closer to the Pacific Ocean.

In some populations, the regression coefficients were similar or larger than those reported above, but were not detected as statistically significant by the regression analysis probably because of small sample size or high variation in the response. We highlighted them in light grey in Tables 1 and 2.

**Table 1:** Relationship between population growth rates and climate indices (NAO = North Atlantic Oscillation index; AO = Arctic Oscillation index; NP = North Pacific index) in Fennoscandia and Taymyr.

		NAO					AO					NP				
Population	Time lag	β	SE	p	R²	O	β	SE	p	R²	O	β	SE	p	R²	O
Norway	t	-0.0022	0.0057	0.7022	0.00	0	-0.0017	0.0068	0.8014	0.00	0	0.0016	0.0023	0.4813	0.01	0
	t-1	**-0.0129**	**0.0055**	**0.0222**	0.10	0	**-0.0159**	**0.0064**	**0.0166**	0.12	0	-0.0005	0.0022	0.8095	0.00	0
	t-2	-0.0046	0.0060	0.4411	0.01	0	-0.0036	0.0070	0.6132	0.01	0	-0.0013	0.0023	0.5620	0.01	0
Sweden	t	0.0001	0.0075	0.9865	0.00	0	-0.0016	0.0080	0.8402	0.00	0	0.0066	0.0080	0.4134	0.02	0
	t-1	-0.0038	0.0094	0.6858	0.00	0	-0.0113	0.0100	0.2664	0.04	0	-0.0024	0.0028	0.3874	0.02	0
	t-2	-0.0041	0.0085	0.6353	0.01	0	-0.0113	0.0090	0.2173	0.05	0	0.0006	0.0028	0.8188	0.00	0
Finland	t	0.0030	0.0117	0.7954	0.00	6	-0.0018	0.0114	0.8748	0.00	3	0.0016	0.0049	0.7458	0.00	6
	t-1	-0.0026	0.0118	0.8292	0.00	6	-0.0024	0.0114	0.8376	0.00	2	0.0068	0.0047	0.1576	0.03	6
	t-2	-0.0153	0.0108	0.1626	0.03	7	-0.0175	0.0112	0.1248	0.04	1	-0.0024	0.0049	0.6334	0.00	6
Hardangervidda	t	0.0170	0.0442	0.7040	0.00	0	0.0213	0.0450	0.6389	0.01	0	0.0136	0.0145	0.3543	0.03	0
	t-1	-0.0036	0.0444	0.9356	0.00	0	-0.0023	0.0459	0.9607	0.00	0	-0.0138	0.0150	0.3663	0.03	0
	t-2	0.0772	0.0397	0.0610	0.11	0	**0.1049**	**0.0423**	**0.0189**	0.17	0	0.0185	0.0137	0.1884	0.06	0
Rondane	t	0.0056	0.0356	0.8753	0.00	0	0.0102	0.0360	0.7792	0.00	0	0.0007	0.0152	0.9631	0.00	0
	t-1	-0.0320	0.0350	0.3698	0.03	0	-0.0211	0.0355	0.5569	0.01	0	0.0055	0.0136	0.6901	0.01	0
	t-2	-0.0547	0.0316	0.0957	0.10	0	-0.0580	0.0326	0.0872	0.11	0	-0.0065	0.0134	0.6305	0.01	0
Snøhetta	t	-0.0192	0.0421	0.6535	0.01	0	-0.0246	0.0416	0.5614	0.02	0	-0.0229	0.0166	0.1851	0.09	0
	t-1	-0.0111	0.0446	0.8059	0.00	0	-0.0101	0.0409	0.8076	0.00	0	0.0000	0.0153	0.9999	0.00	0
	t-2	-0.0192	0.0500	0.7046	0.01	0	0.0057	0.0480	0.9068	0.00	0	0.0142	0.0150	0.3528	0.05	0
Taymyr	t	0.0098	0.0116	0.4112	0.05	1	0.0059	0.0116	0.6198	0.02	1	-0.0026	0.0034	0.4597	0.04	1
	t-1	0.0090	0.0119	0.4605	0.04	1	0.0137	0.0134	0.3226	0.07	1	0.0060	0.0032	0.0833	0.20	1
	t-2	0.0048	0.0155	0.7599	0.01	1	0.0153	0.0160	0.3536	0.06	1	0.0019	0.0039	0.6431	0.02	1

The Norway, Sweden and Finland populations are semi-domesticated, while all other populations are wild. For a detailed explanation of each variable, see the Methods section. Results are based on univariate regression models. D = semi-domesticated; W = wild; β = regression coefficient; SE = standard error; p = p-value; R² = R-squared; O = outliers. Statistically significant results (p < 0.05) are highlighted in bold. Coefficients with a p-value < 0.10 are underlined.

**Table 2:** Relationship between population growth rates and climate indices (NAO = North Atlantic Oscillation index; AO = Arctic Oscillation index; NP = North Pacific index) in the semi-domesticated Russian populations.

			NAO					AO					NP			
Population	Time lag	Predictor	β	SE	p	R²	Predictor	β	SE	p-value	R²	Predictor	β	SE	p-value	R²
Murmansk	t	NAO	0.0344	0.0189	0.0885	0.40	AO	-0.0031	0.0101	0.7611	0.00	NP	0.0013	0.0031	0.6828	0.01
		bSUC	-0.0101	0.0141	0.4852		bSUC	-	-	-		bSUC	-	-	-	
		dSUC	-0.0877	0.0541	0.1248		dSUC	-	-	-		dSUC	-	-	-	
		NAO:bSUC	**-0.0478**	**0.0217**	**0.0424**		AO:bSUC	-	-	-		NP:bSUC	-	-	-	
		NAO:dSUC	0.0119	0.0539	0.8283		AO:dSUC	-	-	-		NP:dSUC	-	-	-	
	t-1	NAO(t-1)	-0.0009	0.0087	0.9167	0.00	AO(t-1)	0.0002	0.0098	0.9827	0.00	NP(t-1)	0.0000	0.0000	0.3986	0.04
		bSUC	-	-	-		bSUC	-	-	-		bSUC	-	-	-	
		dSUC	-	-	-		dSUC	-	-	-		dSUC	-	-	-	
		NAO:bSUC	-	-	-		AO:bSUC	-	-	-		NP:bSUC	-	-	-	
		NAO:dSUC	-	-	-		AO:dSUC	-	-	-		NP:dSUC	-	-	-	
	t-2	NAO(t-2)	-0.0099	0.0100	0.3367	0.05	AO(t-2)	-0.0074	0.0115	0.5254	0.02	NP(t-2)	0.0017	0.0032	0.6138	0.01
		bSUC	-	-	-		bSUC	-	-	-		bSUC	-	-	-	
		dSUC	-	-	-		dSUC	-	-	-		dSUC	-	-	-	
		NAO:bSUC	-	-	-		AO:bSUC	-	-	-		NP:bSUC	-	-	-	
		NAO:dSUC	-	-	-		AO:dSUC	-	-	-		NP:dSUC	-	-	-	
Arkhangelsk	t	NAO	-0.0071	0.0120	0.5615	0.02	AO	-0.0051	0.0135	0.7116	0.01	NP	-0.0004	0.0040	0.9207	0.00
		bSUC	-	-	-		bSUC	-	-	-		bSUC	-	-	-	
		dSUC	-	-	-		dSUC	-	-	-		dSUC	-	-	-	
		NAO:bSUC	-	-	-		AO:bSUC	-	-	-		NP:bSUC	-	-	-	
		NAO:dSUC	-	-	-		AO:dSUC	-	-	-		NP:dSUC	-	-	-	
	t-1	NAO(t-1)	0.0018	0.0114	0.8729	0.00	AO(t-1)	0.0079	0.0131	0.5550	0.24	NP(t-1)	0.0000	0.0000	0.8867	0.00
		bSUC	-	-	-		bSUC	-0.0281	0.0216	0.2117		bSUC	-	-	-	
		dSUC	-	-	-		dSUC	**-0.0580**	**0.0265**	**0.0428**		dSUC	-	-	-	
		NAO:bSUC	-	-	-		AO:bSUC	-	-	-		NP:bSUC	-	-	-	
		NAO:dSUC	-	-	-		AO:dSUC	-	-	-		NP:dSUC	-	-	-	
	t-2	NAO(t-2)	-0.0057	0.0134	0.6747	0.01	AO(t-2)	-0.0017	0.0155	0.9158	0.00	NP(t-2)	0.0107	0.0069	0.1419	0.42
		bSUC	-	-	-		bSUC	-	-	-		bSUC	**19.9820**	**8.8595**	**0.0385**	
		dSUC	-	-	-		dSUC	-	-	-		dSUC	9.4949	11.1143	0.4056	
		NAO:bSUC	-	-	-		AO:bSUC	-	-	-		NP:bSUC	**-0.0198**	**0.0088**	**0.0382**	
		NAO:dSUC	-	-	-		AO:dSUC	-	-	-		NP:dSUC	-0.0095	0.0110	0.4034	
Komi	t	NAO	-0.0223	0.0244	0.3703	0.04	AO	-0.0189	0.0274	0.4995	0.02	NP	-0.0001	0.0083	0.9919	0.00
		bSUC	-	-	-		bSUC	-	-	-		bSUC	-	-	-	
		dSUC	-	-	-		dSUC	-	-	-		dSUC	-	-	-	
		NAO:bSUC	-	-	-		AO:bSUC	-	-	-		NP:bSUC	-	-	-	
		NAO:dSUC	-	-	-		AO:dSUC	-	-	-		NP:dSUC	-	-	-	
	t-1	NAO(t-1)	-0.0256	0.0227	0.2735	0.06	AO(t-1)	-0.0243	0.0263	0.3674	0.04	NP(t-1)	0.0000	0.0000	0.6780	0.01
		bSUC	-	-	-		bSUC	-	-	-		bSUC	-	-	-	
		dSUC	-	-	-		dSUC	-	-	-		dSUC	-	-	-	
		NAO:bSUC	-	-	-		AO:bSUC	-	-	-		NP:bSUC	-	-	-	
		NAO:dSUC	-	-	-		AO:dSUC	-	-	-		NP:dSUC	-	-	-	
	t-2	NAO(t-2)	0.0030	0.0276	0.9134	0.00	AO(t-2)	-0.0101	0.0317	0.7523	0.01	NP(t-2)	-0.0112	0.0084	0.2006	0.08
		bSUC	-	-	-		bSUC	-	-	-		bSUC	-	-	-	
		dSUC	-	-	-		dSUC	-	-	-		dSUC	-	-	-	
		NAO:bSUC	-	-	-		AO:bSUC	-	-	-		NP:bSUC	-	-	-	
		NAO:dSUC	-	-	-		AO:dSUC	-	-	-		NP:dSUC	-	-	-	
Yamal	t	NAO	0.0114	0.0181	0.5363	0.03	AO	0.0287	0.0210	0.1952	0.13	NP	0.0052	0.0058	0.3808	0.06
		bSUC	-	-	-		bSUC	-	-	-		bSUC	-	-	-	
		dSUC	-	-	-		dSUC	-	-	-		dSUC	-	-	-	
		NAO:bSUC	-	-	-		AO:bSUC	-	-	-		NP:bSUC	-	-	-	
		NAO:dSUC	-	-	-		AO:dSUC	-	-	-		NP:dSUC	-	-	-	
	t-1	NAO(t-1)	-0.0053	0.0158	0.7406	0.01	AO(t-1)	0.0014	0.0208	0.9469	0.00	NP(t-1)	**0.0161**	**0.0068**	**0.0319**	0.29
		bSUC	-	-	-		bSUC	-	-	-		bSUC	-	-	-	
		dSUC	-	-	-		dSUC	-	-	-		dSUC	-	-	-	
		NAO:bSUC	-	-	-		AO:bSUC	-	-	-		NP:bSUC	-	-	-	
		NAO:dSUC	-	-	-		AO:dSUC	-	-	-		NP:dSUC	-	-	-	
	t-2	NAO(t-2)	-0.0083	0.0193	0.6739	0.01	AO(t-2)	-0.0114	0.0224	0.6207	0.02	NP(t-2)	0.0030	0.0058	0.6200	0.02
		bSUC	-	-	-		bSUC	-	-	-		bSUC	-	-	-	
		dSUC	-	-	-		dSUC	-	-	-		dSUC	-	-	-	
		NAO:bSUC	-	-	-		AO:bSUC	-	-	-		NP:bSUC	-	-	-	
		NAO:dSUC	-	-	-		AO:dSUC	-	-	-		NP:dSUC	-	-	-	
Sakha	t	NAO	-0.0061	0.0233	0.7960	0.00	AO	0.0162	0.0283	0.5776	0.02	NP	0.0109	0.0070	0.1411	0.15
		bSUC	-	-	-		bSUC	-	-	-		bSUC	-	-	-	
		dSUC	-	-	-		dSUC	-	-	-		dSUC	-	-	-	
		NAO:bSUC	-	-	-		AO:bSUC	-	-	-		NP:bSUC	-	-	-	
		NAO:dSUC	-	-	-		AO:dSUC	-	-	-		NP:dSUC	-	-	-	
	t-1	NAO(t-1)	-0.0331	0.0182	0.0898	0.19	AO(t-1)	-0.0057	0.0265	0.8327	0.00	NP(t-1)	**0.0342**	**0.0066**	**0.0004**	0.82
		bSUC	-	-	-		bSUC	-	-	-		bSUC	**29.6297**	**13.1270**	**0.0476**	
		dSUC	-	-	-		dSUC	-	-	-		dSUC	-19.4601	25.2341	0.4584	
		NAO:bSUC	-	-	-		AO:bSUC	-	-	-		NP:bSUC	**-0.0294**	**0.0130**	**0.0477**	
		NAO:dSUC	-	-	-		AO:dSUC	-	-	-		NP:dSUC	0.0193	0.0250	0.4589	
	t-2	NAO(t-2)	**-0.0538**	**0.0202**	**0.0183**	0.34	AO(t-2)	-0.0493	0.0254	0.0739	0.23	NP(t-2)	0.0040	0.0074	0.6010	0.02
		bSUC	-	-	-		bSUC	-	-	-		bSUC	-	-	-	
		dSUC	-	-	-		dSUC	-	-	-		dSUC	-	-	-	
		NAO:bSUC	-	-	-		AO:bSUC	-	-	-		NP:bSUC	-	-	-	
		NAO:dSUC	-	-	-		AO:dSUC	-	-	-		NP:dSUC	-	-	-	
Chukotka	t	NAO	0.0132	0.0317	0.6847	0.45	AO	0.0600	0.0384	0.1464	0.54	NP	0.0053	0.0079	0.5151	0.70
		bSUC	-0.0174	0.0439	0.6994		bSUC	0.0193	0.0494	0.7035		bSUC	-5.7937	21.6567	0.7945	
		dSUC	**-0.1529**	**0.0508**	**0.0109**		dSUC	**-0.1622**	**0.0466**	**0.0051**		dSUC	**-72.1097**	**28.7528**	**0.0310**	
		NAO:bSUC	-	-	-		AO:bSUC	-	-	-		NP:bSUC	0.0057	0.0215	0.7952	
		NAO:dSUC	-	-	-		AO:dSUC	-	-	-		NP:dSUC	**0.0714**	**0.0285**	**0.0313**	
	t-1	NAO(t-1)	-0.0117	0.0298	0.7008	0.45	AO(t-1)	0.0206	0.0358	0.5761	0.45	NP(t-1)	**0.0250**	**0.0094**	**0.0240**	0.80
		bSUC	-0.0347	0.0475	0.4790		bSUC	-0.0117	0.0492	0.8168		bSUC	14.5300	18.8175	0.4579	
		dSUC	**-0.1388**	**0.0507**	**0.0180**		dSUC	**-0.1536**	**0.0513**	**0.0122**		dSUC	**-88.7460**	**36.1729**	**0.0341**	
		NAO:bSUC	-	-	-		AO:bSUC	-	-	-		NP:bSUC	-0.0144	0.0187	0.4572	
		NAO:dSUC	-	-	-		AO:dSUC	-	-	-		NP:dSUC	**0.0880**	**0.0359**	**0.0342**	
	t-2	NAO(t-2)	-0.0595	0.0302	0.0692	0.22	AO(t-2)	-0.0644	0.0366	0.1020	0.19	NP(t-2)	0.0051	0.0103	0.6269	0.02
		bSUC	-	-	-		bSUC	-	-	-		bSUC	-	-	-	
		dSUC	-	-	-		dSUC	-	-	-		dSUC	-	-	-	
		NAO:bSUC	-	-	-		AO:bSUC	-	-	-		NP:bSUC	-	-	-	
		NAO:dSUC	-	-	-		AO:dSUC	-	-	-		NP:dSUC	-	-	-	
Kamchatka	t	NAO	-0.0387	0.0324	0.2467	0.07	AO	-0.0317	0.0370	0.4029	0.04	NP	0.0062	0.0113	0.5898	0.01
		bSUC	-	-	-		bSUC	-	-	-		bSUC	-	-	-	
		dSUC	-	-	-		dSUC	-	-	-		dSUC	-	-	-	
		NAO:bSUC	-	-	-		AO:bSUC	-	-	-		NP:bSUC	-	-	-	
		NAO:dSUC	-	-	-		AO:dSUC	-	-	-		NP:dSUC	-	-	-	
	t-1	NAO(t-1)	0.0065	0.0314	0.8376	0.00	AO(t-1)	0.0314	0.0343	0.3717	0.04	NP(t-1)	0.0000	0.0000	0.7163	0.01
		bSUC	-	-	-		bSUC	-	-	-		bSUC	-	-	-	
		dSUC	-	-	-		dSUC	-	-	-		dSUC	-	-	-	
		NAO:bSUC	-	-	-		AO:bSUC	-	-	-		NP:bSUC	-	-	-	
		NAO:dSUC	-	-	-		AO:dSUC	-	-	-		NP:dSUC	-	-	-	
	t-2	NAO(t-2)	0.0162	0.0295	0.5882	0.01	AO(t-2)	**0.2519**	**0.0595**	**0.0007**	0.61	NP(t-2)	**0.0513**	**0.0135**	**0.0016**	0.53
		bSUC	-	-	-		bSUC	0.0100	0.0515	0.8479		bSUC	**54.6416**	**18.6231**	**0.0097**	
		dSUC	-	-	-		dSUC	-0.0856	0.0743	0.2673		dSUC	-25.8457	153.2733	0.8682	
		NAO:bSUC	-	-	-		AO:bSUC	**-0.2183**	**0.0675**	**0.0055**		NP:bSUC	**-0.0541**	**0.0185**	**0.0098**	
		NAO:dSUC	-	-	-		AO:dSUC	**-0.2883**	**0.1241**	**0.0346**		NP:dSUC	0.0256	0.1520	0.8683	

Predictor variables in the full regression models are the climate index, a categorical variable representing the period before (bSUC), during (dSUC) and after (aSUC) the collapse of the Soviet Union, and the interaction between the two (represented by a colon symbol). aSUC is the reference category for the categorical variable SUC. If categorical variable and interaction were not statistically significant (p > 0.05), they were removed from the model. For a detailed explanation of each variable, see the Methods section. No outliers were detected. β = regression coefficient; SE = standard error; p = p-value; R² = R-squared. Statistically significant results (p < 0.05) are highlighted in bold. Coefficients with a p-value < 0.10 are underlined.

### Relationship between climate indices and regional temperature and precipitation

Regional mean January-March temperature was positively correlated with the winter NAO and AO indices in most areas. The NP index was positively (but not strongly) correlated to regional mean temperature only in Norway and Chukotka (Table 3). Correlations between regional total precipitation and the NAO and AO indices were much lower and variable. Regional total precipitation was positively correlated to the NP index in most areas of Central and Far Eastern Siberia and negative in one region of Western Russia (Murmansk; Table 3).

**Table 3:** Pearson correlation coefficient values representing the correlation between climate indices (NAO = North Atlantic Oscillation index; AO = Arctic Oscillation index; NP = North Pacific index) and mean annual winter (January–March) temperature and total annual winter precipitation in Fennoscandia and in the range of each Russian reindeer population.

	Temperature			Precipitation		
	NAO	AO	NP	NAO	AO	NP
Norway	**0.78**	**0.78**	**0.20**	**0.42**	**0.52**	-0.11
Sweden	**0.77**	**0.76**	0.14	**0.52**	**0.47**	-0.12
Finland	**0.72**	**0.68**	0.15	NA	NA	NA
Murmansk	**0.69**	**0.65**	0.16	**0.28**	**0.31**	**-0.24**
Arkhangelsk	**0.69**	**0.64**	0.04	**0.30**	**0.37**	-0.13
Komi	**0.61**	**0.54**	-0.10	**0.41**	**0.40**	-0.13
Yamal	**0.58**	**0.53**	-0.13	**0.28**	**0.31**	-0.19
Sakha	**0.56**	**0.61**	-0.01	-0.14	-0.07	**0.29**
Chukotka	-0.04	0.01	**0.14**	**-0.23**	-0.15	**0.31**
Kamchatka	0.04	-0.01	-0.01	-0.15	-0.04	**0.25**
Taymyr	**0.57**	**0.56**	-0.07	0.18	0.23	-0.12

Statistically significant values (p<0.05) are highlighted in **bold**. F = Fennoscandia; R = Russia. Precipitation data for Finland were not available (NA; see Methods section for details).

## Discussion

Trends in population dynamics were very variable across populations, as were the levels of synchrony among population growth rates, and the effects of climate. Previous studies proposed conflicting descriptions of the status of reindeer populations. Some studies suggested that the species is suffering from a global, synchronous decline [49], while others revealed that even populations occupying neighboring ranges experienced very different dynamics in the last decades (examples come from the Yamal Peninsula: [14], and caribou in Alaska: [45]). Bragina et al. [43] did not detect any significant trend (either positive or negative) in the dynamics of wild reindeer in Russia from 1981 to 2010. Our results support the hypothesis of heterogeneous trends and suggest that reindeer are not synchronously declining worldwide as suggested by previous studies (see e.g. [49]). Among the nineteen populations we analyzed, nine populations increased in number during the study period, while seven populations experienced a decrease during either the whole study period or the last two decades (Fig 1). Other than weather, several forces may influence population dynamics of both wild and semi-domesticated reindeer. For example, both groups are harvested by people (even though with different mechanisms and harvesting pressures), vulnerable to predation and diseases, negatively influenced by infrastructure development [50,51], forestry [52,53], mineral extraction [54], and petroleum industry infrastructure [55]. All of these factors shape population dynamics to different degrees together with weather, possibly contributing to the variable patterns we observed. The trends we observed in the wild Russian populations in the last decades should be considered with caution, since counting efforts were reduced after the collapse of the Soviet Union in 1991 and thus population abundances for the last two decades may be underestimated [31]. However, if true population abundances after 1991 were higher than reported here, the increasing long-term trends observed for Taymyr, Lena-Olenek and Sundrun would only be strengthened.

Most of the synchrony in reindeer population dynamics we detected does not seem to be explained by the climate indices we considered. The only populations that might have been synchronously driven by climate are the Sakha and Chukotka semi-domesticated populations. Their dynamics were synchronous (Pearson correlation coefficient = 0.60) and explained by the NP index with the same lagged effect (S3 Table and Table 2). In several other populations climate indices explained growth rates, but the patterns were not linked to synchrony among populations (S3 Table and Tables 1 and 2). Effects were either positive or negative, direct or delayed depending on the population. Those differences may be explained by the different correlation between regional winter weather and climate indices in different regions (Table 3), and by the diversity of effects that climate may have on reindeer populations, i.e. effects on reindeer survival, reproductive success and future reproductive potential of calves (hence the delayed effects; reviewed in [14]).

Part of the synchrony among semi-domesticated Russian populations may be also explained by the socio-economic history of the Soviet Union. For example, the Arkhangelsk, Sakha, Chukotka, and Kamchatka populations experienced a period of stable dynamics during the Soviet Union time, a pronounced decline following the collapse of the Soviet Union (in 1991), and a slow recovery in the last decade (Fig 1). Those patterns were a result of political, social, and economic changes that occurred before and after the Soviet Union collapse [27,41,42]. Klokov [56] concluded that the political context in many regions has been so large as to conceal the impact of factors such as climate change. Our results support this statement by showing how the effects of climate variability on population growth rates differed among the periods before, during and after the collapse of the Soviet Union in many populations (Table 2). Another example of the importance of socio-economics in influencing semi-domesticated populations comes from the Yamal Peninsula. Here, an increasing demand for reindeer products brought by the oil and gas extraction industries has supported the economy of reindeer husbandry, despite the negative impacts that those industries exert on reindeer pastures (Fig 1, but see also [9,10]). However, the situation is highly dynamic, even in regions like the Yamal Peninsula, characterized by a steadily increasing reindeer population for most of the last century [57,58]. Severe ice crusts during winter 2013–14 resulted in the deaths of ca. 61000 animals out of a population of ca. 300000 on Yamal Peninsula (unpublished data), indicating that the increased severity and extent of such events projected by Bartsch et al. [14] has come to pass and is having a significant impact. Further studies are needed to assess the effect of local weather on the Russian reindeer populations and to determine how local and regional weather interact with human pressures in shaping their population dynamics.

We did not detect any correlation between distance among population ranges and synchrony (Fig 2). This result confirms the importance of drivers other than migration between populations and climate in the dynamics of the studied populations. Indeed, the link between climate indices and growth rates was apparent in only a few populations (Tables 1 and 2). That result can be explained in several ways: i) predator pressure, disease outbreaks, human disturbances or management strategies might have overridden climatic impacts [59–61]; ii) different populations may have reacted to the same climatic drivers in different ways, depending on the pastures they used (shown for Finnish herds by Kumpula and Colpaert [60] and in Norway by Tveera et al. [61]); or iii) local weather may have been a stronger driver of population dynamics than the climate patterns described by the NAO, AO, and NP indices. Those indices correlate with regional and local temperature and precipitation with strong spatial variability (see Table 3 and e.g. [32,34,47]). Therefore, they may be good predictors of population dynamics in some areas, but not in others. Stenseth and Mysterud [62] argued that climate indices predict life history traits of mammals better than local weather, as they combine changes in several weather aspects in time and space. For example, in Finland semi-domesticated reindeer mortality is explained both by the NAO and AO indices, but not by local weather [18]. However, climate indices failed to explain variability in growth rates in many of the populations we analyzed. Similarly, Post and Forchhammer [37] found that the relationship between the two-year lagged NAO index and reindeer population dynamics varies across Russia. Recently, van de Pol et al. [63] showed that climate indices may give spurious results on the inter- or intraspecific variation in response to climate over a large area, due to their weak link to local weather in some regions. This statement supports the lack of effect in some of the populations reported here. Aanes et al. [36] suggested that the AO index may be better at predicting the ecological patterns of the Arctic compared to the NAO, which may have smaller-scale effects on regional weather. However, in our study the dynamics of only two populations (Norway and Hardangervidda) were explained by the AO index. Extreme weather events that are short lived, but have disproportionally strong impacts on survival, such as ice-crust formation events, may be better predictors of reindeer population dynamics, but data on these events are often difficult to obtain [64–66].

The scale of study can also produce diverse results. As mentioned above, Helle and Kojola [18] detected a relationship between the NAO and AO indices and reindeer mortality, i.e. one component of population dynamics, in a Finnish herd. Similarly, Helle and Kojola [17] found a positive relationship between the AO index and semi-domesticated reindeer abundance in some counties of Norway, Sweden, and Finland, while we failed to detect any influence of NAO and AO indices on the Swedish and Finnish semi-domesticated populations at the country level. The different spatial scale between those studies and ours (i.e. a single herd/county level *vs*. the whole country) may explain the different results we obtained. Moreover, the negative relationship between the NAO index and reindeer population dynamics in Sweden between 1996 and 2008 (detected by Hobbs et al. [16]) seems to be restricted to that time period since it was not apparent in our analysis of a longer time series. By pooling data at the country level, we masked some of the events that drove reindeer population dynamics at small spatial or temporal scales (such as, e.g., the large changes in subsidies that were introduced in the ‘70s and ‘80s in Norway [67]). However, the aim of our study was to detect the power of climate in overriding those events and driving reindeer population dynamics in a synchronous way across a large spatial and temporal scale.

In this study, populations were not divided based on count method used to collect abundance data and accuracy of the data. However, we are confident that our results are not confounded by small inaccuracies in the data, as the ones detected for the Norwegian, Finnish and Taymyr populations (see Methods section). Small counting errors do not undermine our conclusions because we were not interested in quantifying the magnitude of synchrony and effect of climate on reindeer numbers, but rather to detect general patterns. Similar considerations were made by Tyler [68]. Nevertheless, caution should be adopted when interpreting the trends of the wild Russian populations, due to the reduced sample size available for those populations (S2 Table) and the possible inaccuracy in the data (see Methods section).

Our study has shown that generalizations about the future abundance of this species should be avoided, especially as large fluctuations are common in Arctic ungulate population dynamics over the long-term [68,69]. Effects of global climate change may not be apparent in all reindeer populations yet, but may become apparent in the future [70]. Understanding how population dynamics are coupled to, or independent from, historic climatic forcing cannot conclusively contribute to future predictions of climate change effects on the viability of a given species, especially if predicted changes in climate will go beyond the previously experienced range of variability [71,72]. We thus cannot conclude if the climate in the future will have similar effects as the ones we observed in this analysis. Moreover, the effects of climate change will continue to act together with and be influenced by human disturbances and biotic interactions [73].

In some cases, human disturbances may have a stronger impact than climate on reindeer population dynamics. This view is supported by Rees et al. [74], who suggested that the vulnerability to climate change of reindeer husbandry in Eurasia is comparatively small in relation to the influence of regional socio-economic dynamics (see also [9,10]), as well as by Syroechkovskiĭ [31], who emphasized the importance of poaching in shaping the dynamics of wild reindeer in Russia and Siberia. We therefore point to the high significance of human decision making, particularly in Eurasia where most of the reindeer populations are semi-domesticated and thus highly dependent on herders’ management decisions, and where industrial developed areas have direct and indirect impacts on essential habitats for both wild and semi-domesticated reindeer [9,75,76].

In the future, the persistence of both wild and semi-domesticated reindeer populations may confer resilience to the species and determine its survival, with semi-domesticated populations possibly being a source of individuals to wild populations. Conservation efforts of wild populations should address issues such as poaching and loss of habitat, as these may interact with climate change in affecting population dynamics. The semi-domesticated populations that have synchronous dynamics will have a higher chance of collapsing or going extinct [73]. Synchronously fluctuating reindeer populations in Far Eastern Siberia may thus be vulnerable under future global changes that negatively influence reindeer survival. Those populations should be the target of focused conservation efforts in order to preserve the traditional indigenous practices that characterize the area and the fundamental role of reindeer in Arctic ecosystems.

## Supporting Information

S1 Table Data source and sampling method of reindeer abundance time series. (DOCX)

S2 Table Number of years of available population abundance (N) and period of available data (Y, years) for each of the 19 reindeer populations we analyzed. (DOCX)

S3 Table Pearson correlation coefficient values indicating synchrony among reindeer population growth rates. (DOCX)
