# Supplementary material for: Long-Term Trends and Role of Climate in the Population Dynamics of Eurasian Reindeer
Source: PLoS One. 2016 Jun 30;11(6):e0158359. doi: 10.1371/journal.pone.0158359 (PMC4928808; doi:10.1371/journal.pone.0158359)
Supplement: S1 Table — (DOCX) [file pone.0158359.s001.docx]

**S1 Table**. **Data source and sampling method of reindeer abundance time series.**

| **Population** | **Source** | **Sampling method** |
| --- | --- | --- |
| Norway, semi-domesticated | Before 1980: A. Ermala (pers. comm., Finnish Game and Fisheries Research Institute) and NINA Rapport 672 (http://www.nina.no/archive/nina/PppBasePdf/rapport/2011/672.pdf); after 1980: Reindrifts kommisionen (http://www.reindrift.no) | Annual counts done by herders during round-ups before calving in March [for details see [1](#_ENREF_1),[2](#_ENREF_2)] |
| Sweden, semi-domesticated | Sametinget (Sami Parliament); Jämtland, Västerbotten, and Norrbotten County Administration Boards; [[3](#_ENREF_3)]; SOU 1983:67; [[4](#_ENREF_4),[5](#_ENREF_5)]; [[6](#_ENREF_6)] | Annual counts done by herders during round-ups for winter slaughter in December [[for details see 7](#_ENREF_7)] |
| Finland, semi-domesticated | Before 1970: Kortesalmi [[8](#_ENREF_8)] complemented with A. Ermala (pers. comm.); after 1970: Paliskuntainyhdistys (Finnish Reindeer Herders Association, Rovaniemi, Finland; [www.paliskunnat.fi](http://www.paliskunnat.fi)) | Annual counts done by herders during round-ups for winter slaughter between October and February. |
| Norway, wild | Jordhøy [[9](#_ENREF_9)] and Solberg et al. [[10](#_ENREF_10)]; Hardangervidda before1990: Skogland [[11](#_ENREF_11)], available from the Global Population Dynamics Database, Imperial College London, UK | Hardangervidda before 1990: counts conducted from aircraft in July [[for details see 11](#_ENREF_11)]. Hardangervidda after 1990, Rondane and Snøhetta: minimum count of population abundance conducted from helicopter or airplane in July (O. Strand, pers. comm.). |
| Finland, wild | Ministry of Agriculture and Forestry 2007 (<http://www.rktl.fi>); Kojola et al. [[12](#_ENREF_12)] | Counts performed from helicopter in March [for details see [12](#_ENREF_12),[13](#_ENREF_13)]. |
| Russia, semi-domesticated | Years 1941-2000: Russian Reindeer Herding Report (1999) and Syroechkovskiĭ [[14](#_ENREF_14),[15](#_ENREF_15)]; years 2001-2009: Minsel'khoz, Reindeer Herder Union, Russia, except year 2007 (sel'khozperepis) | Official numbers reported by the herders to the respective agricultural administration in winter. |
| Russia, wild | CARMA-website (http://www.carmanetwork.com); Ministry of Nature Protection in Sakha (Yakutia) (<http://www.nature.ykt.ru/zhiv_mir.htm>, assessed on November 20^th^ 2009); [[16](#_ENREF_16)]; [[15](#_ENREF_15)]; [[17](#_ENREF_17)]; [[18](#_ENREF_18)]; [[19, pp. 150-152](#_ENREF_19)]; Kolpashchikov (pers. comm. for Taymyr in 2009) | Population abundances refer to July-August counts conducted from aircraft [for details see [20](#_ENREF_20),[21](#_ENREF_21),[22](#_ENREF_22)]. |

# References

1. Tveraa T, Fauchald P, Gilles Yoccoz N, Anker Ims R, Aanes R, et al. What regulate and limit reindeer populations in Norway? Oikos. 2007; 116: 706-715.

2. Weladji RB, Holand Ø. Global climate change and reindeer: effects of winter weather on the autumn weight and growth of calves. Oecologia. 2003; 136: 317-323.

3. Sverige Statistiska Centralbyrån. Historisk statistik för Sverige II. Sweden: Kungl. Boktryckeriet; 1959. *English title:* Historic statistics for Sweden II.

4. SOU. Renndringens ekonomi. Stockholm, Sweden: Allmanna forlaget; 1983:67. *English title:* The economy of the reindeer husbandry.

5. Kungl. Utrikesdepartamentet. Betänkande - avgivet av svensk-norska renbeteskommissionen av 1964. Stockholm, Sweden: Kungl. Boktryckeriet P. A. Norstedt & Söner; 1967. *English title:* Report - given by the Swedish-Norwegian reindeer Commission 1964.

6. Svenska Samernas Riksförbund, Jordbruksverket, Sveriges Lantbruks Universitet, Centralbyrån S. Svensk rennäring. Halmstad, Sweden: Bulls Tryckeriaktiebolag; 1999. *English title:* Swedish reindeer husbandry.

7. Hobbs NT, Andrèn H, Persson J, Aronsson M, Chapron G. Native predators reduce harvest of reindeer by Sami pastoralists. Ecol Appl. 2012; 22: 1640-1654.

8. Kortesalmi J. Poronhoidon synty ja kehitys Suomessa. Helsinki, Finland: Suomalaisen kirjallisuuden seura; 2007. *English title:* The origins and development of reindeer husbandry in Finland.

9. Jordhøy P. Villreinen i Rondane – Sølnkletten. Kunnskapstatus og leveområde. NINA Rapport 339. Trondheim, Norway; 2008. *English title:* Caribou in Rondane - Sølnkletten. Knowledge status and habitat.

10. Solberg E, Strand O, Veilberg V, Andersen R, Heim M, et al. Hjortevilt 2008 – Årsrapport fra overvåkningsprogrammet for hjortevilt. NINA Rapport 477. Trondheim, Norway; 2009. *English title:* Cervids 2008 - Annual report from the monitoring program for cervids.

11. Skogland T. Density dependence in a fluctuating wild reindeer herd; maternal vs. offspring effects. Oecologia. 1990; 84: 442-450.

12. Kojola I, Tuomivaara J, Heikkinen S, Heikura K, Kilpeläinen K, et al. European wild forest reindeer and wolves: endangered prey and predators. Ann Zool Fenn. 2009; 46: 416-422.

13. Anonymous. Suomen metsäpeurakanna hoitosuunnitelma. Finland: Ministry of Agriculture and Forestry; 2007. *English title:* Finnish forest reindeer-bear management plan.

14. Syroechkovskiĭ EE. Wild and domestic reindeer in Russia: Population trends in current social and economic conditions. Moscow, St. Petersburg: Ethno-ecological Studies; 2000.

15. Syroechkovskiĭ EE. Wild reindeer. Washington, D.C., USA: Smithsonian Institution Libraries; 1995.

16. Pavlov B, Kolpashchikov L, Zyryanov V. Taymyr wild reindeer populations: management experiment. Rangifer. 1993: 381-384.

17. Yakushkin G, Michurin L, Pavlov B, Zyryanov V. Numbers and migrations of wild reindeer in Taymyr; 1970; Moscow, Russia. pp. 335 - 339.

18. Kolpashchikov L The reindeer population of Taymyr (Biological basis of management and sustainable use). Institute Problem Ekologii i Evolutsii, Moscow, Russia. 2000.

19. Baskin L, Danell K. Ecology of ungulates: a handbook of species in Eastern Europe and Northern and Central Asia. Berlin Heidelberg New York: Springer-Verlag; 2003.

20. Baskin LM. Number of wild and domestic reindeer in Russia in the late 20th century. Rangifer. 2005; 25: 51-57.

21. Kolpasсhikov L, Makhailov V, Russell DE, Monitoring H. The role of harvest, predators, and socio-political environment in the dynamics of the Taimyr wild reindeer herd with some lessons for North America. Ecol Soc. 2015; 20: 9.

22. Safronov V. Ekologiya i ispol'zovanie dikogo severnogo olenya v Yakutii. Yakutsk, Russian Federation: Yakutskii filial gosudarstvennogo ychrezhdeniya izdatel'stvo Sibirskoe otdelenie Rossiiskoi akademii nauk; 2005. *English title:* Ecology and the use of wild reindeer in Yakutia.
