# Supplementary material for: Long-Term Trends and Role of Climate in the Population Dynamics of Eurasian Reindeer
Source: PLoS One. 2016 Jun 30;11(6):e0158359. doi: 10.1371/journal.pone.0158359 (PMC4928808; doi:10.1371/journal.pone.0158359)
Supplement: S2 Table — (DOCX) [file pone.0158359.s002.docx]

**S2 Table. Number of years of available population abundance (N) and period of available data (Y, years) for each of the 19 reindeer populations we analyzed.** F = Fennoscandia, R = Russia.

|  |  |  | **N** | **Y** | | |
| --- | --- | --- | --- | --- | --- | --- |
| **Semi-domesticated** | **F** | Norway | 52 | 1945-2011 | | |
|  |  | Sweden | 36 | 1946-2010 | | |
|  |  | Finland | 66 | 1941-2008 | | |
|  | **R** | Murmansk | 23 | | 1941-2009 |  |
|  |  | Arkhangelsk | 23 | | 1941-2009 |  |
|  |  | Komi | 23 | | 1941-2009 |  |
|  |  | Yamal | 17 | | 1941-2009 |  |
|  |  | Sakha | 17 | | 1941-2009 |  |
|  |  | Chukotka | 17 | | 1941-2009 |  |
|  |  | Kamchatka | 24 | | 1941-2009 |  |
| **Wild** | **F** | Hardangervidda | 34 | 1952-2008 | | |
|  |  | Rondane | 29 | 1970-2007 | | |
|  |  | Snøhetta | 22 | 1975-2008 | | |
|  |  | Kainuu | 12 | 1993-2013 | | |
|  |  | Suomenselkä | 8 | 1992-2013 | | |
|  | **R** | Lena-Olenek | 15 | 1959-2009 | | |
|  |  | Yana-Indigirka | 12 | 1963-2002 | | |
|  |  | Sundrun | 13 | 1963-2012 | | |
|  |  | Taymyr | 22 | 1959-2009 | | |
