# Supplementary material for: Long-Term Trends and Role of Climate in the Population Dynamics of Eurasian Reindeer
Source: PLoS One. 2016 Jun 30;11(6):e0158359. doi: 10.1371/journal.pone.0158359 (PMC4928808; doi:10.1371/journal.pone.0158359)
Supplement: S3 Table — (DOCX) [file pone.0158359.s003.docx]

**S3 Table. Pearson correlation coefficient values indicating synchrony among reindeer population growth rates.** Statistically significant (p < 0.05) values are highlighted in **bold**. Positive, significant correlations > 0.40 are highlight in grey. Time series of population growth rates were paired and reduced to include only years in which data were available for both populations in the pair. F = Fennoscandia; R = Russia.

|  |  |  | **Semi-domesticated** | | | | | | | | | | **Wild** | | | |
| --- | --- | --- | --- | --- | --- | --- | --- | --- | --- | --- | --- | --- | --- | --- | --- | --- |
|  |  |  | **F** | | | **R** | | | | | | | **F** | | | **R** |
|  |  |  | Norway | Sweden | Finland | Murmansk | Arkhangelsk | Komi | Yamal | Sakha | Chukotka | Kamchatka | Hardangervidda | Rondane | Snøhetta | Taymyr |
| **Semi-domesticated** | **F** | Norway | 1.00 |  |  |  |  |  |  |  |  |  |  |  |  |  |
|  |  | Sweden | **0.45** | 1.00 |  |  |  |  |  |  |  |  |  |  |  |  |
|  |  | Finland | **0.40** | 0.10 | 1.00 |  |  |  |  |  |  |  |  |  |  |  |
|  | **R** | Murmansk | 0.26 | -0.31 | -0.39 | 1.00 |  |  |  |  |  |  |  |  |  |  |
|  |  | Arkhangelsk | 0.37 | 0.15 | 0.12 | 0.20 | 1.00 |  |  |  |  |  |  |  |  |  |
|  |  | Komi | -0.37 | 0.07 | 0.34 | -0.03 | **0.52** | 1.00 |  |  |  |  |  |  |  |  |
|  |  | Yamal | 0.63 | -0.63 | -0.48 | **0.73** | 0.24 | -0.32 | 1.00 |  |  |  |  |  |  |  |
|  |  | Sakha | **0.55** | 0.25 | **0.67** | -0.04 | **0.40** | 0.25 | -0.25 | 1.00 |  |  |  |  |  |  |
|  |  | Chukotka | **0.54** | 0.16 | 0.12 | **0.37** | **0.46** | -0.04 | 0.16 | 0.66 | 1.00 |  |  |  |  |  |
|  |  | Kamchatka | 0.37 | 0.33 | **0.22** | 0.20 | 0.28 | -0.12 | 0.09 | 0.61 | **0.84** | 1.00 |  |  |  |  |
| **Wild** | **F** | Hardangervidda | 0.21 | 0.23 | 0.19 | -0.19 | -0.18 | -0.36 | -0.72 | 0.39 | 0.18 | 0.00 | 1.00 |  |  |  |
|  |  | Rondane | 0.14 | 0.02 | 0.16 | -0.05 | 0.27 | 0.10 | 0.15 | 0.26 | 0.16 | -0.15 | -0.01 | 1.00 |  |  |
|  |  | Snøhetta | -0.39 | -0.01 | -0.28 | 0.12 | 0.31 | 0.37 | **-0.90** | NA | 0.41 | -0.05 | 0.03 | -0.23 | 1.00 |  |
|  | **R** | Taymyr | -0.27 | -0.14 | **-0.32** | NA | NA | NA | NA | NA | NA | 0.35 | -0.27 | -0.27 | -0.25 | 1.00 |
